# Short Communication: Enterotoxin Genes and Antibiotic Susceptibility of *Bacillus cereus* Isolated from Garlic Chives and Agricultural Environment

**DOI:** 10.3390/ijerph191912159

**Published:** 2022-09-26

**Authors:** Jieun Jung, Hyeonsuk Jin, Seungmi Seo, Myeongin Jeong, Boeun Kim, Kyoungyul Ryu, Kwangkyo Oh

**Affiliations:** 1 Functional Food Division, Department of Agro-Food Resources, National Institute of Agricultural Sciences, Rural Development Administration, Wanju-gun 55365, Jeolloabuk-do, Korea; 2 Microbial Safety Division, Department of Agro-food Safety and Crop Protection, National Institute of Agricultural Sciences, Rural Development Administration, Wanju-gun 55365, Jeollabuk-do, Korea

**Keywords:** agricultural environment, antibiotic resistance, *Bacillus cereus*, enterotoxin, garlic chive

## Abstract

This study aims to investigate the enterotoxin profiles and antibiotic susceptibility of *Bacillus cereus* isolated from garlic chives and environmental samples. A total of 103 *B. cereus* isolates were used to identify enterotoxin genes, including *hblA, hblC, hblD, nheA, nheB*, and *nheC*. The hemolysin BL enterotoxin complex (*hblACD*) was detected in 38 isolates (36.9%), and the non-hemolytic enterotoxin complex (*nheABC*) was detected in 8 (7.8%) isolates. Forty-five isolates (43.7%) had *hblACD* and *nheABC* genes. *B. cereus* was resistant to β-lactam antibiotics and susceptible to non-β-lactam antibiotics. However, some *B. cereus* strains showed intermediate resistance to β-lactam and non-β-lactam antibiotics. *B. cereus* isolated from garlic chives showed intermediate resistance to cefotaxime (7.7%), rifampin (15.4%), clindamycin (30.8%), erythromycin (7.7%), and tetracycline (7.7%). *B. cereus* isolates from the agricultural environment were moderately resistant to cefotaxime (18.9%), rifampin (15.6%), clindamycin (12.2%), erythromycin (4.4%), and tetracycline (5.6%). Moreover, *B. cereus* isolates from garlic chives and cultivation environments could change their antibiotic resistance profile from susceptible to intermediate-resistant to rifampin, clindamycin, erythromycin, and tetracycline and exhibit multidrug resistance. These results indicate that continuous monitoring of *B. cereus* contamination in the produce and agricultural environment might be needed to ensure the safety of consuming fresh vegetables.

## 1. Introduction

The current food trends caused by the COVID-19 pandemic have renewed the interest in healthy lifestyles motivating the consumption of healthy foods [1]. Fresh produce provides vitamins, minerals, and fibers [2]. Consumption of fresh vegetables is considered to prevent obesity, cardiovascular diseases, and osteoporosis [2,3]. However, leafy vegetables are among the foods most associated with disease outbreak [4]. The foodborne outbreaks are caused by contaminated vegetables and related pathogenic bacteria such as *Bacillus cereus*, *Salmonella*, and *Escherichia coli* O157:H7 [1,5]. Vegetables are often consumed directly or only with minimal processing that does not eliminate pathogenic bacteria [5]. Food poisoning outbreaks caused by food contaminated with *B. cereus* included 120 cases from 2003 to 2022 in Korea [6].

*B. cereus* is a gram-positive, spore-forming, facultative aerobe motile rod and an opportunistic human pathogen that belongs to the *B. cereus* species group [7,8,9]. This group consists of the eight species *B. cereus*, *B. mycoides*, *B. pseudomycoides*, *B. thuringiensis*, *B. weihenstephanensis*, *B. anthracis*, *B. cytotoxicus,* and *B. toyonensis* [9]. *B. cereus* causes food spoilage and food poisoning in humans [8]. Food poisoning caused by *B. cereus* is of two types: diarrheal and emetic. The diarrheal type is caused by the production of heat-labile enterotoxins produced during the vegetative growth of *B. cereus* in the small intestine, including hemolysin BL (HBL), non-hemolytic enterotoxin (NHE), single protein enterotoxin cytotoxin K, and enterotoxin FM, whereas the emetic type is caused by cereulide produced during the growth of *B. cereus* cells in food [9,10,11]. Foods often related to diarrheal food poisoning include meat products, soups, vegetables, sauces, and dairy products, while those related to the emetic food poisoning are mainly rice and pasta [12]. Some *B. cereus* strains cause hospital acquired infections; however, the occurrence of these infections caused by *B. cereus* is low, but the mortality is high, regardless of aggressive treatment with antibiotics [13]. *B. cereus* produces β-lactamases, and it is resistant to β-lactam antibiotics, including the third generation cephalosporins. However, *B. cereus* is susceptible to clindamycin, aminoglycosides, chloramphenicol, vancomycin, and erythromycin [14].

Garlic chives (Korean leek, *Allium tuberosum* Rottler) belong to the family *Alliaceae* and include garlic and onions, which are some of the most commonly used vegetable ingredients in Korean dishes [15]. Garlic chives are rich in nutrients such as vitamins, carbohydrates, minerals, and cellulose [16]. A study reported that the level of *B. cereus* contamination in garlic chives was 1.30 to 5.08 log CFU/g [17]. Since garlic chives are cultivated in contact with the soil, contaminated soil can cause cross-contamination with *B. cereus* in garlic chives [17].

The distribution of *B. cereus* in plants and cultivated environments has been reported in previous studies. However, the number of studies on plants and cultivated environments, especially composts and irrigation water, is insignificant. Additionally, there are few studies pertaining to the characteristics of the enterotoxin profile and antibiotic resistance of *B. cereus* isolated from cultivated environments. Therefore, the purpose of this study was to investigate the pattern of enterotoxin genes and antibiotic resistance of *B. cereus* isolated from garlic chives and agricultural environment including soil, compost, and irrigation water.

## 2. Materials and Methods

### 2.1. Bacterial Strains

Garlic chives and agricultural environment including soil, composts, and irrigation water were collected from garlic chive farms in Korea. *B. cereus*, which was isolated by MYP agar from samples, was collected and stocked in our previous study [18]. The β-hemolysis and groEL gene of *B. cereus* were identified using the blood agar culture method and polymerase chain reaction (PCR). In total, 13 garlic chive samples, 67 soil samples, 17 compost samples, and 6 irrigation water samples were studied in this study [18].

### 2.2. Detection of Enterotoxin Genes

The isolates were streaked on tryptic soy agar (TSA) and incubated at 28 °C for 18 to 24 h. The DNA templates were extracted using DNA extraction kit for the PCR assay. PCR amplification was conducted with a 20 μL reaction mixture consisting of AccuPower PCR premix (Bioneer, Daejeon, Korea), 20 to 50 ng of DNA template, and 10 pmol of each primer using a thermal cycler (C1000TM Thermal Cycler, BIO-RAD, CA, USA). The primer pairs used for amplifying the *hblACD* and *nheABC* genes were prepared as described by Park et al. [14]. Amplification reactions were performed as described by Park et al. [14] with modifications. The template DNA was preheated to 94 °C for 7 min. The *hblA* gene was amplified for 35 cycles of 45 s at 94 °C for denaturation, 45 s at 58 °C for annealing, and 45 s at 72 °C for extension, followed by a final extension at 72 °C for 7 min. The PCR conditions for the *hblC* and *hblD* genes consisted of 35 cycles of 30 s at 94 °C for denaturation, 30 s at 54 °C for annealing, and 30 s at 72 °C for extension. The PCR conditions for the *nheA, nheB,* and *nheC* genes consisted of 35 cycles of 30 s at 94 °C for denaturation, 30 s at 55 °C for annealing, and 30 s at 72 °C for extension. The PCR products were electrophoresed on a 2% agarose gel. *B. cereus* ATCC 14579 was used as the control.

### 2.3. Antibiotic Susceptibility Testing

Antibiotic susceptibility of *B. cereus* was evaluated according to the method described by the Clinical and Laboratory Standards Institute (CLSI) [19]. The antimicrobial agents tested and their concentrations were as follows: penicillin (10 U), oxacillin (1 μg), cefotaxime (30 μg), cefoxitin (30 μg), imipenem (10 μg), gentamicin (10 μg), streptomycin (10 μg), rifampicin (5 μg), trimethoprim-sulfamethoxazole (25 μg), vancomycin (30 μg), clindamycin (2 μg), erythromycin (15 μg), linezolid (30 μg), chloramphenicol (30 μg), tetracycline (30 μg), and ciprofloxacin (5 μg). The susceptibility of *B. cereus* to each antimicrobial agent was measured, and the results were interpreted in accordance with the criteria provided by the CLSI. *Staphylococcus aureus* ATCC 29213 was selected as the control organism.

## 3. Results and Discussion

### 3.1. Distribution of Enterotoxin Genes in B. cereus from Different Sources

Diverse patterns of enterotoxin gene distribution were identified in *B. cereus* isolated from garlic chives and agricultural environments. Garlic chives had 4 patterns, soil had 11 patterns, compost had 6 patterns, and irrigation water had 1 pattern (Table 1). HBL and NHE complexes (*hblA* + *hblC* + *hblD* and *nheA* + *nheB* + *nheC*) were 23.1% (pattern G1), HBL complex was 61.5% (pattern G2), and NHE complex was 15.4% (pattern G3, G4) in garlic chives. In soil, HBL and NHE complexes were 47.8% (pattern S1), HBL complex was 32.8% (pattern S2-4), and NHE complex was 9.0% (pattern S5, S8). *B. cereus* isolated from soil has one or two hemolytic enterotoxin genes and two non-hemolytic enterotoxin genes on four different patterns (10.0%) and exhibits *hblCD* genes on one pattern (1.5%). In compost, HBL and NHE complexes were 23.5% (pattern C1) and HBL complex was 47.1% (pattern C2). *B. cereus* isolated from irrigation water showed 100% of HBL and NHE complexes (pattern W1). *B. cereus* isolated from compost has two hemolytic enterotoxin genes and one or two non-hemolytic enterotoxin genes on two patterns (11.8%) and exhibits one or two non-hemolytic enterotoxin genes on two patterns (17.6%).

HBL, a three-component hemolysin, consisting of a binding component (B, *hblA*) and lytic components (L1&L2, *hblD*, and *hblC*) and exhibiting enterotoxin activity, has been purified and characterized [20]. HBL complex has maximal hemolytic and cytotoxic activities [7]. NHE is a pore-forming toxin consisting of two lytic elements, *nheA* and *nheB*, and the protein *nheC* [21]. Since HBL and NHE are tripartite toxins, in both cases the three components are necessary to produce the active toxin [22]. HBL and NHE are considered the main virulence factors of *B. cereus* [14]. *B. cereus* is found in the ground, dust, or on different foods. Virulence or enterotoxin gene has been isolated from foods, clinical, soil, and environment samples [23]. *B. cereus* isolated from green leaves or vegetables such as garlic chives, bell peppers, perilla leaf, and romaine lettuce had high detection rates of the *hblACD* and *nheABC* genes [14]. Amor et al. [24] reported that diverse patterns of enterotoxin distribution of *B. cereus* were detected from fresh-cut vegetables in Tunisia; 20% HBL complex, 60% *hblC* + *hblD* gene, and 100% NHE complex [24]. In the present results, *B. cereus* isolated from garlic chives had 7.7% of *hblC* + *hblD* gene and 38.5% of NHE complex. A previous study [25] reported that *B. cereus s.l.* isolated from fresh vegetable samples such as cucumbers, carrots, herbs, salad leaves, and ready-to-eat mixed salads had various patterns; 91.2% *hblDA*, 73.5% *nheAB*, and 53.7% *hblDA* + *nheAB* complex. *B. cereus* strains isolated from Mexican chili powder were found to be positive for the *hblC* and *nheA* genes [26]. However, in the present study, 100, 92.3, 38.5, and 7.7% of *B. cereus* isolates from garlic chives were positive for *hblD*, *hblC*, *nheA*, and *hblDA* + *nheABC* complex (Table 1). Senesi and Ghelardi [12] reported that HBL was secreted by approximately 43% and NHE was produced by almost 100% of *B. cereus* strains isolated from environment and/or food. However, our study demonstrated that *B. cereus* isolated from garlic chives, soil, compost, and irrigation water secreted 80.6% the HBL complex and 51.5% NHE complex.

### 3.2. Antibiotic Susceptibility of B. cereus

**Figure 1:**
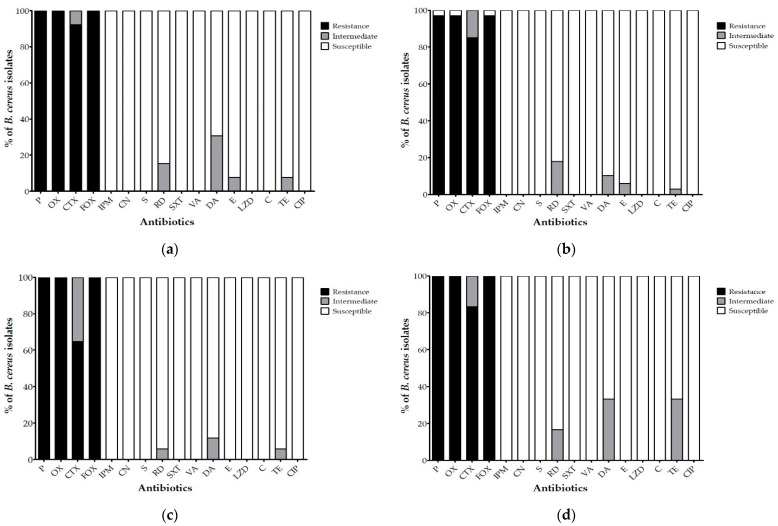
Antibiotic susceptibility of *B. cereus* isolated from garlic chives (**a**), soil (**b**), compost (**c**), and irrigation water (**d**) was determined by disk diffusion method. P, penicillin; OX, oxacillin; CTX, cefotaxime; FOX, cefoxitin; IPM, imipenem; CN, gentamicin; S, streptomycin; RD, rifampin; SXT, trimethoprim-sulfamethoxazole; VA, vancomycin; DA, clindamycin; E, erythromycin; LZD, linezolid; C, chloramphenicol; TE, tetracycline; CIP, ciprofloxacin.

The antibiotic resistance of *B. cereus* isolates to diverse antimicrobial agents is shown in Figure 1. Overall, *B. cereus* isolates were resistant to penicillin, oxacillin, cefotaxime, and cefoxitin, but were susceptible to imipenem. One isolate from garlic chives showed intermediate resistance to cefoxitin (Figure 1a). Two isolates from soil were susceptible to penicillin, oxacillin, and cefoxitin, and 10 isolates from soil had intermediate resistance to cefotaxime (Figure 1b). Six isolates from compost and one isolate from irrigation water possessed intermediate resistance to cefotaxime (Figure 1c,d). *B. cereus* isolated from garlic chives, soil, compost, and irrigation water was susceptible to non-β-lactam antibiotics, including gentamicin, streptomycin, rifampin, trimethoprim-sulfamethoxazole, vancomycin, clindamycin, erythromycin, linezolid, chloramphenicol, tetracycline, and ciprofloxacin. However, some isolates had intermediate resistance to antimicrobial agents such as rifampin, clindamycin, erythromycin, and tetracycline. Furthermore, *B. cereus* isolated from garlic chives showed intermediate resistance to rifampin (15.4%), clindamycin (30.8%), erythromycin (7.7%), and tetracycline (7.7%) (Figure 1a). *B. cereus* isolated from soil showed intermediate resistance to rifampin (17.9%), clindamycin (10.4%), erythromycin (6%), and tetracycline (3%) (Figure 1b). *B. cereus* isolated from compost had intermediate resistance to rifampin (5.9%), clindamycin (11.8%), and tetracycline (5.9%) (Figure 1c). *B. cereus* from irrigation water had intermediate resistance to rifampin (16.7%), clindamycin (33.3%), and tetracycline (33.3%) (Figure 1d). *B. cereus* ATCC 14579 showed resistance to penicillin, oxacillin, and cefoxitin, intermediate resistance to cefotaxime and rifampin, and susceptibility to 11 antibiotics.

A previous study reported that *B. cereus* strains isolated from raw vegetables such as garlic chives, bell peppers, perilla leaf, and romaine lettuce exhibited resistance to penicillin, cefotaxime, tetracycline, clindamycin, and rifampin [14]. *B. cereus* strains isolated from perilla leaf showed resistance to penicillin, resistance and susceptibility to oxacillin, resistance, intermediate resistance, and susceptibility to rifampin, and susceptibility to imipenem [27]. *B. cereus* isolated from clinical patients and foods, including dairy products, salad, rice, and infant food, had resistance to some glycopeptides, aminoglycosides, tetracycline, and carbapenems [28]. Our results were consistent with the patterns reported in previous studies. We found that *B. cereus* isolated from garlic chives exhibited the resistance against penicillin, oxacillin, and cefotaxime, the intermediate resistance against rifampin, clindamycin, and tetracycline, and susceptibility to imipenem. However, the pattern of tetracycline observed in our study (intermediate resistance and susceptibility) differed from that reported previously. Jensen et al. [29] reported that *B. cereus* group isolated from farm soil had penicillin, erythromycin, and streptomycin resistance. However, in our study, *B. cereus* isolated from soil showed resistance to penicillin, intermediate resistance to erythromycin, and susceptibility to streptomycin and erythromycin. *B. cereus* produces β-lactamase and is resistant to β-lactam antibiotics including third generation cephalosporins [13]. Most *B. cereus* isolates in this study showed resistance to penicillin, oxacillin, cefotaxime, and cefoxitin. It has been reported that *B. cereus* isolated from grassland soil was resistant to penicillin, sulbactam+ampicillin, trimethoprim-sulfamethoxazole, and oxacillin [30]. *B. cereus* is generally susceptible to clindamycin, aminoglycosides, chloramphenicol, vancomycin, and erythromycin [31]. Luna et al. [32] reported that *B. cereus* isolated from the environment and soil showed intermediate resistance or resistance to clindamycin and resistance to erythromycin. The variations in antibiotic resistance profiles observed in this study can be explained by the fact that environmental conditions can induce stress in bacteria, thereby impacting bacterial susceptibility to antimicrobials [33]. When bacteria are exposed to environmental stress, they may undergo genotypic and phenotypic changes, which may subsequently change their antibiotic resistance profiles [13].

## 4. Conclusions

The present study revealed the various pattern of enterotoxin profiles in *B. cereus* isolated from garlic chives and the agricultural environment (soil, compost, and irrigation water) in Korea. *B. cereus* isolates had resistance to penicillin, oxacillin, cefotaxime, and cefoxitin, and intermediate resistance to cefotaxime, rifampin, clindamycin, erythromycin. The results of the present study indicate the potential of *B. cereus* in garlic chives and the agricultural environment to cause diarrhea syndrome. Additionally, *B. cereus* strains exhibited multidrug resistance and the diversity of antibiotic resistance profiles showed that it changed from susceptibility to intermediate resistance or resistance to intermediate resistance and susceptibility. Therefore, it needs the intensive monitoring of garlic chives and agricultural environment to protect consumer health from food poisoning and antibiotic multi-resistance.

## Figures and Tables

**Table 1:** *B. cereus* isolated from garlic chives and environment had diverse patterns of enterotoxin genes distributions.

Sample	No. of Isolates (%)	Toxin Genes								
		Pattern	*hblA*	*hblC*	*hblD*	*hblACD* ^1^	*nheA*	*nheB*	*nheC*	*nheABC* ^2^
Garlic chives	3/13 (23.1)	G1	+	+	+	+	+	+	+	+
	8/13 (61.5)	G2	+	+	+	+	—	+	+	—
	1/13 (7.7)	G3	+	—	+	—	+	+	+	+
	1/13 (7.7)	G4	—	+	+	—	+	+	+	+
Soil	32/67 (47.8)	S1	+	+	+	+	+	+	+	+
	7/67 (10.4)	S2	+	+	+	+	+	—	+	—
	3/67 (4.5)	S3	+	+	+	+	+	—	—	—
	12/67 (17.9)	S4	+	+	+	+	—	+	+	—
	5/67 (7.5)	S5	+	—	+	—	+	+	+	+
	1/67 (1.5)	S6	+	—	+	—	+	+	—	—
	2/67 (3.0)	S7	+	—	+	—	—	+	+	—
	1/67 (1.5)	S8	—	+	+	—	+	+	+	+
	2/67 (3.0)	S9	—	+	+	—	+	—	+	—
	1/67 (1.5)	S10	—	+	+	—	—	—	—	—
	1/67 (1.5)	S11	—	—	+	—	+	—	+	—
Compost	4/17 (23.5)	C1	+	+	+	+	+	+	+	+
	8/17 (47.1)	C2	+	+	+	+	—	+	+	—
	1/17 (5.9)	C3	+	—	+	—	+	—	—	—
	1/17 (5.9)	C4	—	+	+	—	+	—	+	—
	2/17 (11.8)	C5	—	—	—	—	+	—	+	—
	1/17 (5.9)	C6	—	—	—	—	—	—	+	—
Irrigation water	6/6 (100.0)	W1	+	+	+	+	+	+	+	+

^1^ hblACD, hblA + hblC + hblD. ^2^ nheABC, nheA + nheB + nheC.

## Data Availability

Not applicable.
